# IFN regulatory factor 3 of golden pompano and its NLS domain are involved in antibacterial innate immunity and regulate the expression of type I interferon (IFNa3)

**DOI:** 10.3389/fimmu.2023.1128196

**Published:** 2023-02-02

**Authors:** Yun Sun, Zhenjie Cao, Panpan Zhang, Caoying Wei, Jianlong Li, Ying Wu, Yongcan Zhou

**Affiliations:** ^1^ State Key Laboratory of Marine Resource Utilization in South China Sea, Hainan University, Haikou, China; ^2^ Collaborative Innovation Center of Marine Science and Technology, Hainan University, Haikou, China; ^3^ Hainan Provincial Key Laboratory for Tropical Hydrobiology and Biotechnology, College of Marine Science, Hainan University, Haikou, China

**Keywords:** IRF3, *Trachinotus ovatus*, antimicrobial immunity, nuclear localization signal, IFNa3

## Abstract

**Introduction:**

The transcription factor interferon regulatory factor 3 (IRF3) plays an important role in host defence against viral infections. However, its role during bacterial infection in teleosts remains unclear. In the present study, we evaluated the antibacterial effects of *Trachinotus ovatus* IRF3 (TroIRF3) and how it regulates type I interferon (IFN).

**Methods:**

Subcellular localisation experiments, overexpression, and quantitative real-time PCR (qRT-PCR) were performed to examine the nuclear localisation signal (NLS) of TroIRF3 and its role in the antibacterial regulatory function of TroIRF3. We assessed the binding activity of TroIRF3 to the IFNa3 promoter by luciferase reporter assay.

**Results and Discussion:**

The results showed that TroIRF3 was constitutively expressed at high levels in the gill and liver. TroIRF3 was significantly upregulated and transferred from the cytoplasm to the nucleus after *Vibrio harveyi* infection. By overexpressing TroIRF3, the fish were able to inhibit the replication of *V. harveyi*, whereas knocking it down increased bacterial replication. Moreover, the overexpression of TroIRF3 increased type I interferon (IFNa3) production and the IFN signalling molecules. The NLS, which is from the 64–127 amino acids of TroIRF3, contains the basic amino acids KR74/75 and RK82/84. The results proved that NLS is required for the efficient nuclear import of TroIRF3 and that the NLS domain of TroIRF3 consists of the key amino acids KR74/75 and RK82/84. The findings also showed that NLS plays a key role in the antibacterial immunity and upregulation of TroIFNa3 induced by TroIRF3. Moreover, TroIRF3 induces TroIFNa3 promoter activity, whereas these effects are inhibited when the NLS domain is deficient. Overall, our results suggested that TroIRF3 is involved in the antibacterial immunity and regulation of type I IFN in *T. ovatus* and that the NLS of TroIRF3 is vital for IRF3-mediated antibacterial responses, which will aid in understanding the immune role of fish IRF3.

## 1 Introduction

Innate immunity is the first line of defence against invading viruses and bacteria. The innate antiviral mechanism in mammals involves interferon (IFN), which induces the expression of IFN-relative genes (1–3). IFN can be divided into two major categories based on its cysteine residues. In all teleost fish lineages, type I IFNs contain two cysteine residues; however, type II IFNs contain four cysteine residues in a few species (4–7). In both vertebrates and invertebrates, interferon regulatory factors (IRFs) regulate type I and II IFN transcriptional activation (8).

Thus far, nine IRF family members have been found in mammals, 10 in birds and 11 in fish (9). The N-terminal DNA binding domain (DBD), consisting of five tryptophan repeats, is highly homologous between IRF members. The DBD forms a helix-turn-helix structure that binds specifically to IFN-stimulated response element (ISRE) sequences in the promoters of IFNβ and IFN-stimulated genes (ISGs) (10). Except for IRF1 and IRF2, the C-terminal of each IRF has an IRF association domain (IAD), which is used to interact with other IRFs and other factors (11–13). IRF3 and IRF7 contain a serine-rich region (SRD) at the C-terminus that controls their transcriptional activities when phosphorylated by viruses (11–14). Two transcription factors, IRF3 and IRF7 control the expression of type I IFNs (15, 16). The antiviral effects of several innate immune receptors, including retinoic acid-inducible gene I (RIG-I)-like receptors (RLRs), toll-like receptors (TLRs) and DNA sensors, are mediated by IRF3 (17–19). All TLRs in mammals (except TLR3) transduce signals through the MyD88-dependent pathway using molecules such as the TNFR-associated factor 6 (TRAF6) (17, 20–22). In general, pathogen invasion initiates an immune response by activating the immune signalling pathways. As a member of the IRF family, IRF3 plays an important role in immune signalling. Following bacterial infection, IRF3 is responsible for controlling the expression of IFNs and ISGs (23). ISGs include ISG15, MAX interactor I (MXI) and Viperin1 (24).

Accurate cellular localization plays a crucial role in the effective function of most signaling proteins, the ability of IRF3 to translocate into the nucleus is essential for its biological activity. Nuclear localization signals (NLSs) and nuclear export signals (NESs) are intrinsic targeting signals required to facilitate the shuttle between the nucleus and the cytoplasm for proteins larger than 45 kDa (25). IRF3 accumulates in the cytoplasm under resting conditions and adopts an auto-inhibited conformation, which is mediated by the NES. When infected, IRF3 enters the nucleus *via* NLS after being phosphorylated and dimerized at its C-terminus. IRF3 and the coactivator CBP/p300 form a complex and then bind the IFN-α/β promoters and the ISRE sequences of the targeted genes (14, 26, 27). Several studies have found that bacterial products, such as lipopolysaccharide (LPS), can activate toll-like receptors to induce type I IFN expression through IRF3 signalling (28–31). However, the role of IRF3 in bacterial infection is less understood.

The characteristics and functions of IRF3s have been reported in several species of teleost fish, such as *Epinephelus coioides*, *Cynoglossus semilaevis*, *Carassius auratus* L., *Salmo salar*, *Larimichthys crocea*, and *Paralichthys olivaceus* (32–37). In a previous study, red-spotted grouper nervous necrosis virus replication was significantly decreased in *E. coioides* when IRF3 was overexpressed (32). For *C. auratus* L., fish IFN or polyinosinic-polycytidylic acid (poly I:C) induced the phosphorylation of IRF3 and translocation from the cytoplasm to the nucleus. In addition, *C. auratus* L. IRF3 overexpression induced IFN production, which subsequently triggered ISG transcription *via* signal transducer and activator of transcription 1 (STAT1) (33). Moreover, viral and bacterial stimulation significantly induced *C. semilaevis* IRF3 (34). Although some studies have investigated IRF3 in fish, its function, particularly its role in antibacterial immunity, remains largely unknown.

*Vibrio harveyi* is a gram-negative opportunistic pathogenic bacterium of many commercially farmed fish species (38, 39). *Trachinotus ovatus* (golden pompano) is one of the most economically important aquaculture species in China that suffers from *V. harveyi*, resulting in serious economic losses (40). IFNa3 is a member of type I IFNs in *T. ovatus*. Previous study demonstrated that IRF1, IRF2, IRF5, and IRF7 positively regulate IFNa3 expression in *T. ovatus* (41, 42). The purpose of this study was to assess the antibacterial immune effects of IRF3 in *T. ovatus* (TroIRF3) and its role in the regulation of IFNa3. Based on these investigations, we further examine the NLS in TroIRF3 and its role in the host antibacterial immune response induced by TroIRF3.

## 2 Materials and methods

### 2.1 Fish, cell lines and bacteria

*T. ovatus* were purchased from a commercial fish farm in Hainan, China. Before the experiment, the fish were acclimated at 26°C in a flow-through water system for one week. All animal experiments conducted during this study were approved by the Animal Care and Use Committee at Hainan University. Human embryonic kidney (HEK293T) cells were cultured at ambient CO_2_ and 37°C in Dulbecco’s modified Eagle’s medium (DMEM, USA), the DMEM contained 1% penicillin and streptomycin (Pen Strept) and 10% foetal bovine serum (FBS). Golden pompano snout (GPS) cells were generously donated by the South China Sea Institute of Oceanology Chinese Academy of Sciences. The GPS cells were grown at 26°C with Leibovitz’s L-15 medium containing 10% FBS (Gibco, USA) (42, 43). *V. harveyi* was grown at 28°C in Luria-Bertani broth (38).

### 2.2 TroIRF3 cloning

Based on the transcriptome data, the primers TroIRF3-F/TroIRF3-R were used to clone the open reading frame (ORF) of TroIRF3 by polymerase chain reaction (PCR) amplification (**Table S1**). Sequencing was performed following gel purification of the PCR products into the pEASY^®^-T1Simple Vector (Transgen, Beijing, China). A homology search of the TroIRF3 protein sequence was conducted using BLAST programs on NCBI (http://www.ncbi.nlm.nih.gov/blast). Signal peptide prediction was performed using SignalP 5.1. Thereafter, DNAMAN (Lynnon Biosoft, USA) was used to align the amino acid sequences. SWISS-MODEL was used to predict the TroIRF3’s tertiary structure. A phylogenetic analysis was performed using neighbour-joining methods through MEGA 6.0.

### 2.3 Expression profiles of TroIRF3 in normal tissues and when challenged with *V. harveyi*

A total of 11 tissues were obtained from 15 healthy *T. ovatus* (blood, head kidney, liver, intestine, spleen, gill, muscle, skin, brain, stomach and heart). To avoid the affection of the individual difference of fish, the fish tissues were mixed aseptically into a sample with five identical samples. The tissue samples were stored at −80°C until RNA extraction was performed. The *T. ovatus* were divided into two groups: control and bacterial challenge. The challenge group received 0.1 ml of *V. harveyi* (2 × 10^6^ CFU/ml), while the control group received the same volume of PBS. At 6, 9, 12 and 24 hours post-injection (hpi), 15 individuals in each group were randomly sacrificed, and the liver, spleen and head-kidney were sampled. Five identical tissues from each group were mixed aseptically to form a sample. The total RNA was extracted from the harvested samples using the Total RNA Extraction Kit (Promega, China). The cDNA was synthesised using the Reverse Transcription System (Promega, China). An SYBR ExScript qRT–PCR kit (LS2062, Promega, China) was used to perform quantitative real-time PCR (qRT-PCR) on a qTOWER3 Real-Time PCR (qPCR) system (Analytik Jena, Germany). The internal reference gene was *β-2 microglobulin* (*B2M*), and the relative expression levels of *TroIRF3* were calculated using the 2^^-ΔΔCT^ method (44). The primers are summarised in **Table S1**.

### 2.4 TroIRF3 overexpression and *V. harveyi* infection *in vivo*

The eukaryotic expression vector pTroIRF3 was constructed for TroIRF3 overexpression *in vivo*. TroIRF3 was cloned into the pCN3’s *Sma* I site and transformed into *Escherichia coli*, DH5α. pTroIRF3 was extracted using the Endo Free Plasmid Kit (Tiangen, China). The fish (weight 15.2 ± 3.1 g) were randomised into three groups (N=12), with each receiving a 0.1 ml injection of pTroIRF3 (150 μg/ml), pCN3 (empty vector, 150 μg/ml) or PBS (control). To detect the expression level of TroIRF3, the liver, spleen and head-kidney of three fish from each group were collected after five days of intramuscular injection of plasmids. After the isolation of total RNA and cDNA synthesis, TroIRF3 expression was detected by qRT-PCR with primer TroIRF3-RT-F/R (**Table S1**). *B2M* was used as the internal reference gene. *V. harveyi* (10^5^ CFU/fish) was injected into the remaining fish. The liver, spleen and head-kidney of three fish in each group were homogenised at 6, 9 and 12 hpi for bacterial count analysis.

### 2.5 TroIRF3 knockdown and *V. harveyi* infection *in vivo*

The small interfering RNA (siRNA) of TroIRF3 was synthesised using the T7 RiboMAX™ Express RNAi System (Promega, USA). The primers used for siRNA synthesis are listed in **Table S1**. A total of 36 fish were randomly divided into three groups of 12 each and injected with 0.1 ml of PBS (control), siRNA-C (control, 15 μg/fish) or siRNA-TroIRF3 (15 μg/fish). At 12 h after intramuscular injection of siRNA, three fish were sacrificed for liver, spleen and head-kidney collection. Then, qRT–PCR was performed to detect the TroIRF3 expression levels. The rest of the fish were infected with *V. harveyi* (10^5^ CFU/fish). The liver, spleen and head-kidney of three fish in each group were homogenised at 6, 9, and 12 hpi for bacterial count analysis.

### 2.6 Subcellular localisation

The eukaryotic expression vector pTroIRF3-N3 was created by cloning TroIRF3’s ORF into the *Bgl* II and *Sal* I sites of pEGFP-N3. Subcellular localisation experiments were performed to examine TroIRF3 localisation in the cell with or without *V. harveyi* infection. The cells were seeded on 24-well plates and grown overnight, and then Lipofectamine 2000 (Invitrogen, USA) was used to transfect pEGFP-N3 or pTroIRF3-N3 into the GPS cells. At 48 h post-transfection, the cells transfected with pTroIRF3-N3 were treated with 0.1 ml PBS (control) or *V. harveyi* (1×10^5^ CFU/ml) for 6 h. The GPS cells were washed, stained and visualised with fluorescence microscopy (Leica, Wetzlar, Germany) after fixing in 4% paraformaldehyde.

Different PCR fragments of TroIRF3 were inserted into *Bgl* II and *Sal* I sites of pEGFP-N3 vector to constructe TroIRF3-GFP truncation mutants: pTroIRF3-(△NES)-N3, pTroIRF3-1-165-N3, pTroIRF3-1-417-(△NES)-N3, pTroIRF3-64-466-(△NES), or pTroIRF3-127-466-(△NES). Various plasmids were transfected into the GPS cells to detect the NLSs of TroIRF3. The cells were fixed, washed, stained and visualised under fluorescence microscopy at 48 h post-transfection.

Two TroIRF3 variants (KR74/75NG and RK82/84LQ) were designed to further detect the NLSs of IRF3 within the aa 64–127 region. The PCR fragments of KR74/75NG and RK82/84LQ were inserted into *Bgl* II and *Sal* I sites of pEGFP-N3 vector to construct two recombinant plasmids: pTroIRF3-KR74/75NG-N3 and pTroIRF3-RK82/84LQ-N3. The primers used are listed in **Table S1**. The GPS cells were transfected with plasmids (pTroIRF3-KR74/75NG-N3 or pTroIRF3-RK82/84LQ-N3). After 48 h, the cells were treated with 0.1 ml *V. harveyi* (1×10^5^ CFU/ml) or PBS (control) for 6 h. Afterwards, the cells were fixed, washed, stained and visualised under fluorescence microscopy.

### 2.7 Role of NLSs sites in immune response *in vivo*

#### 2.7.1 Role of NLSs in the antibacterial regulatory function of TroIRF3

To further investigate the role of these basic amino acids in TroIRF3’s antibacterial regulation, two recombinant plasmids (pTroIRF3-KR74/75NG and pTroIRF3-RK82/84LQ) were constructed by inserting the variants into the *Sma* I sites of the pCN3 vectors. The fish were randomised into four groups (12 fish per group) and injected with PBS (control), pTroIRF3 (15 μg/fish), pTroIRF3-KR74/75NG (15 μg/fish), or pTroIRF3- RK82/84LQ (15 μg/fish), respectively. After five days, the liver, spleen and head-kidney of three fish from each group were collected to detect the expression level of TroIRF3 or it mutants. The remaining fish were intraperitoneally infected with *V. harveyi* (2×10^6^ CFU/fish). The liver, spleen and head-kidney of three fish in each group were homogenised at 6, 9 and 12 hpi for bacterial count analysis.

#### 2.7.2 Role of NLSs in immune gene expression induced by TroIRF3

Twelve fish were randomised into four groups and injected with PBS (control), pTroIRF3, pTroIRF3-KR74/75NG or pTroIRF3-RK82/84LQ, respectively. After five days of injection, the spleens were collected, followed by RNA isolation and cDNA synthesisation. The relative transcription level of *IFNa3*, *MXI*, *TRAF6*, *Viperin1* and mitochondrial antiviral signalling (*MAVS*) was detected by qRT-PCR. The primers are listed in **Table S1**.

### 2.8 Luciferase reporter assay

The TroIRF3 binding site in the promoter of TroIFNa3 was evaluated by luciferase reporter assay. Three truncation mutants of the TroIFNa3 promoter (−1649 to +1, –896 to +1 and −722 to +1) were amplified and inserted into the *Kpn* I and *Xho* I restriction sites of the pGL4-basic luciferase reporter plasmid (Promega, USA) to obtain the recombinant plasmid pGL4-TroIFNa3-p1, pGL4-TroIFNa3-p2 and pGL4-TroIFNa3-p3. The primers used are summarised in **Table S1**. Lipofectamine 2000 (Invitrogen, USA) was used to cotransfect pRL-TK (0.025 μg), pGL4-TroIFNa3-p1/p2/p3 (0.25 μg) and pTroIRF3 (0.25 μg) into the HEK293T cells. pRL-TK was used as an internal control. After 36 h of transfection, the firefly and renilla luciferase activities were measured using a dual-luciferase reporter assay system (Promega, USA). The activity ratio between the firefly luciferase and the renilla luciferase was used to calculate the luciferase activity. The effect of *V. harveyi* stimulation on the TroIFNa3 promoter activation mediated by TroIRF3 was detected. After 24 h of transfection, the transfected HEK293T cells were treated with PBS or *V. harveyi* (1×10^2^ CFU/ml) for 12 h. Luciferase activity was measured in the lysed cells as described above.

Luciferase reporter assay was performed to detect whether the activation of the TroIFNa3 promoter was dependent on the pTroIRF3 concentration. The HEK293T cells were cotransfected with pRL-TK (0.025 μg), pGL4-TroIFNa3-p2 (0.25 μg) and different concentrations of pTroIRF3 (0, 0.01, 0.05, 0.1 or 0.2 μg). After 24 h, the transfected HEK293T cells were treated with *V. harveyi* (1×10^2^ CFU/ml) for 12 h. Luciferase activity was measured in the lysed cells as described above.

Luciferase reporter assay was performed to detect the role of the basic amino acids (KR74/75 and RK82/84) in the TroIRF3-triggered TroIFNa3-p2 activation. The HEK293T cells were cotransfected with pGL4-TroIFNa3-p2, wild-type TroIRF3 or its variants (pTroIRF3-KR74/75NG or pTroIRF3-RK82/84LQ) and pRL-CMV. After 24 h, the cells were treated with *V. harveyi* (1×10^2^ CFU/ml) for 12 h. Afterwards, the relative luciferase activity was tested as described above.

### 2.9 Statistical analysis

All data were analysed by SPSS 16.0 (SPSS, IL, USA) and GraphPad Prism 5 (GraphPad, CA, USA). The statistical significance level of this study was set at 0.05.

## 3 Results

### 3.1 Cloning and sequence analysis of TroIRF3

**Figure 1:**
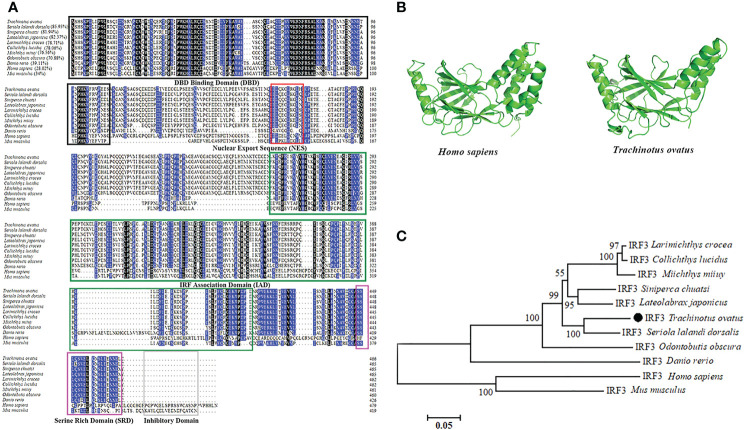
Sequence characteristics of TroIRF3. **(A)** Alignment of amino acid sequences from TroIRF3 and other IRF3 homologs. Identities between TroIRF3 and the compared sequences are shown in brackets. The background color of amino acid residues corresponds to the conservation degree (blue > 75%, black = 100%). The conserved DBD domain is boxed in black, the conserved IAD domain is boxed in green, the conserved SRD domain is boxed in purple, and the conserved inhibitory domain is boxed in gray. The nuclear export sequences (NES) are marked with the red box. **(B)** Analysis of the SWISS-MODEL prediction for TroIRF3 (SWISS-MODEL Template ID: 5jek.2) compared to human IRF3. **(C)** Phylogenetic analysis of TroIRF3. MEGA 6.0 was used to construct a phylogenetic tree based on neighbor-joining (NJ). The sequences of TroIRF3 genes used in this study were obtained from GenBank, a list of GenBank accession numbers can be found in **Table S2**.

The full length of TroIRF3 cDNA contains a 1398 bp ORF encoding 465 amino acids (GenBank Accession No. AWY04222.1). In theory, it has a molecular mass of 52 kDa and an isoelectric point of 4.89. According to the amino acid alignments, TroIRF3 contains three conserved domains, namely, DBD, IAD and SRD (**Figure 1A**). Among the selected IRF3 sequences, TroIRF3 was closest to that of *Seriola lalandi dorsalis* (85.95%), followed by those of *Siniperca chuatsi* and *Lateolabrax japonicus* (81.94% and 82.37%, respectively) (**Figure 1A**). According to the SWISS-MODEL online software, TroIRF3 has a similar three-dimensional structure to human IRF3 (**Figure 1B**). In addition, the IRF3 from fish and mammals were located in two separate branches, while TroIRF3 was inserted in the fish cluster. TroIRF3 shows a close evolutionary relationship with *S. lalandi dorsalis* IRF3 (**Figure 1C**).

### 3.2 Expression pattern of TroIRF3 with or without bacterial challenge

**Figure 2:**
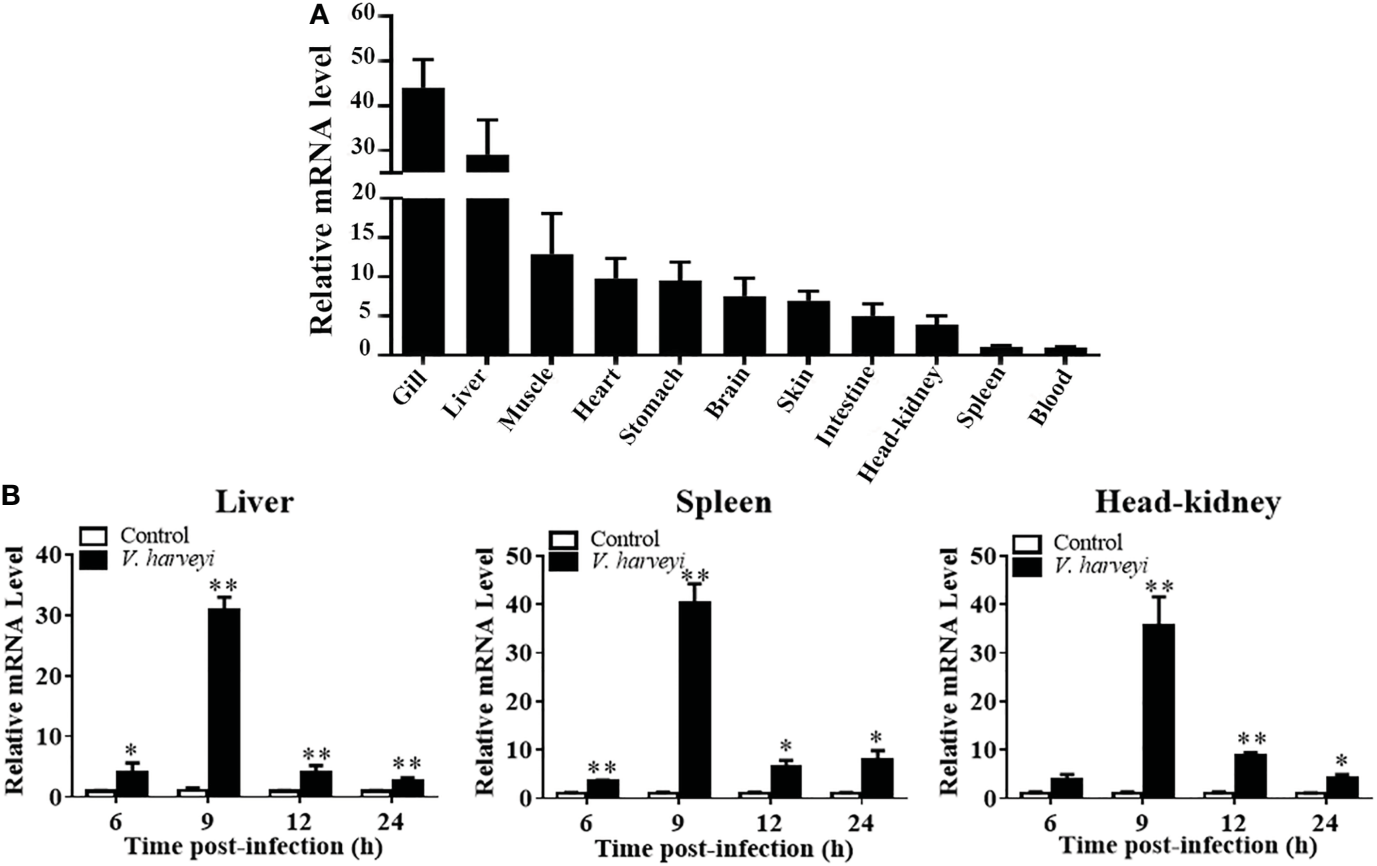
Expression profiles of TroIRF3 in normal tissues and when challenged with *V. harveyi*. **(A)** TroIRF3 expression levels in different tissues. **(B)** Expression of TroIRF3 in the liver, spleen, and head-kidney in response to the *V. harveyi* challenge. At every time point, control fish were set to have an average expression level of 1. Error bars display means ± SD (N = 3). N, indicates parallel experiments. *P < 0.05, **P < 0.01.

The expression level of *TroIRF3* was detected in 11 normal tissues (spleen, head-kidney, skin, muscle, gill, heart, liver, brain, stomach, blood and intestine) by qRT-PCR. *TroIRF3* was constitutively distributed in all the tissues examined. *TroIRF3* expression was high in the gill and liver but low in the spleen and blood (**Figure 2A**).

The level of *TroIRF3* expression following *V. harveyi* infection was detected using qRT-PCR (**Figure 2B**). The level of *TroIRF3* expression in the liver was increased from 6 hpi to 24 hpi (4.1-, 30.9-, 4.1-, and 2.7-folds at 6, 9, 12, and 24 hpi, respectively), the expression level of *TroIRF3* in the spleen was increased from 6 hpi to 24 hpi (3.5-, 40.2-, 6.5-, and 7.8-folds at 6, 9, 12, and 24 hpi, respectively), and the expressions of *TroIRF3* in the head-kidney were increased from 9 hpi to 24 hpi but manifested no difference at 6 hpi (35.5-, 8.8-, and 4.0-folds at 9, 12, and 24 hpi, respectively). Based on these results, TroIRF3 may contribute to antibacterial immunity.

### 3.3 Overexpression of TroIRF3 decreases *V. harveyi* replication *in vivo*

**Figure 3:**
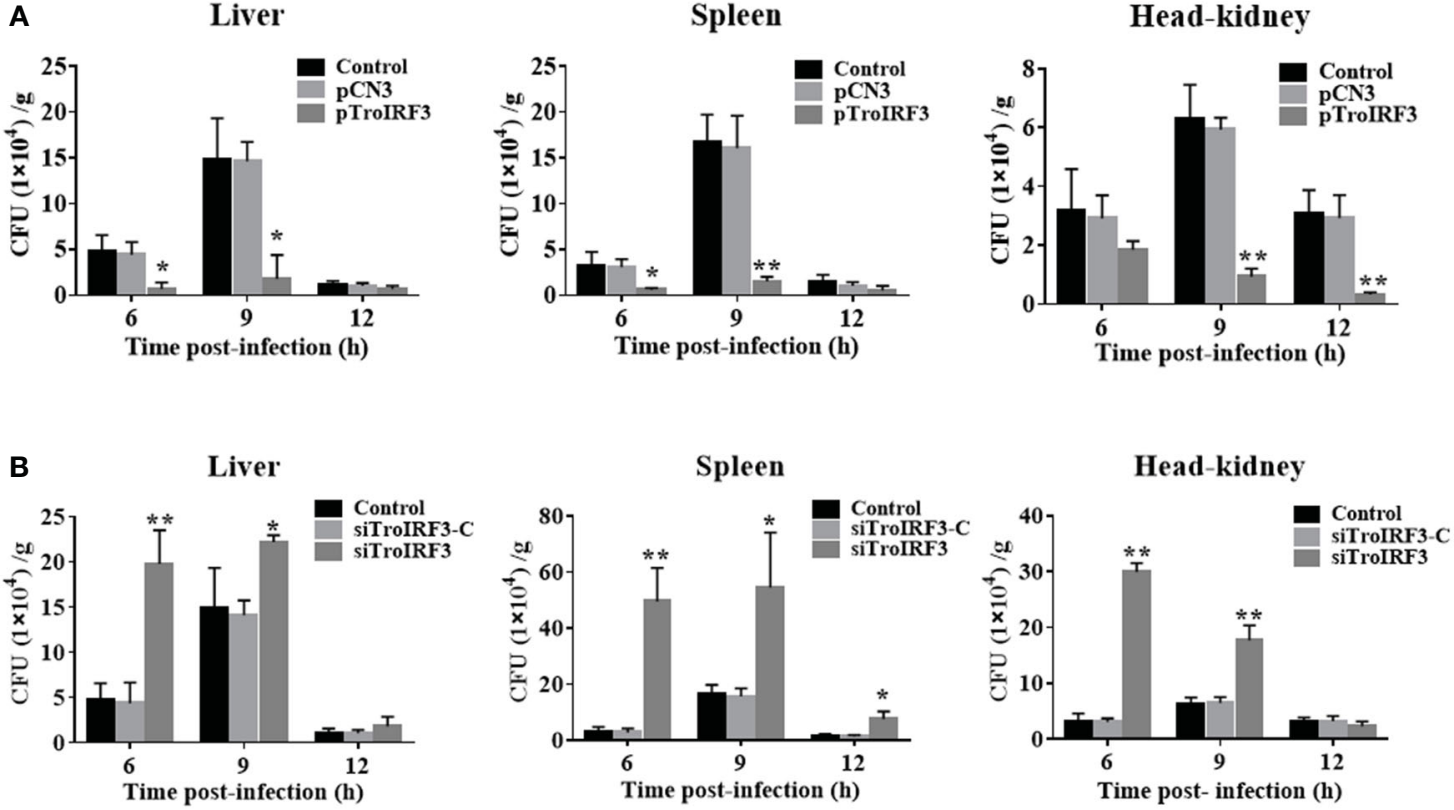
Effects of TroIRF3 on bacterial infection. **(A)** At five days after injection with PBS (Control), pCN3, and pTroIRF3, *T. ovatus* was infected with *V. harveyi*, and their liver, spleen, and head-kidney bacteria were determined at 6, 12, and 24 hpi. **(B)** *T. ovatus* was injected with siTroIRF3, siTroIRF3-C, or PBS (Control) for 12 h, then infected with *V. harveyi*, and their liver, spleen, and head-kidney bacteria were determined at 6, 12, and 24 hpi. The data are shown as the means ± SD (N =3), N, the number of fish used at each time point per group. *P < 0.05, **P < 0.01.

To clarify TroIRF3’s role in antibacterial immunity, we assessed the effects of TroIRF3 overexpression on the fish’s antibacterial ability. An increase in *TroIRF3* expression was observed in fish treated with pTroIRF3, indicating the overexpression of TroIRF3 (**Figure S1A**). Compared with the control group, the number of bacteria in the tissues decreased in the TroIRF3-overexpressing fish (**Figure 3A**). The bacterial load in the liver was significantly decreased in the TroIRF3-overexpressing fish compared with the control fish by 7.1- and 8.3-fold at 6 and 9 hpi, respectively. The pattern of bacterial load in the spleen of the pTroIRF3-overexpressing fish was similar to that in the liver, which decreased significantly with 5.0- and 10.9-fold at 6 and 9 hpi, respectively. Compared with the control fish, TroIRF3 overexpression significantly reduced the number of bacterial colonies in the head-kidney by 6.8- and 9.6-fold at 9 and 12 hpi, respectively. No significant difference in bacterial loads was observed between the empty vector-injected fish and the control fish, which showed that the vector had no effect on the fish’s antibacterial immune responses.

### 3.4 Knockdown of TroIRF3 increases *V. harveyi* replication *in vivo*

The effects of TroIRF3 on antibacterial immune responses were further investigated, and TroIRF3 was knocked down using siRNA administration. The *TroIRF3* gene was decreased *in vivo* using siRNA (**Figure S1B**). Compared with the control fish, the number of bacteria in the tissues obviously increased in the siTroIRF3-treated fish (**Figure 3B**). Compared with the control fish, the TroIRF3 knockdown fish exhibited an increased number of bacteria in the liver by 4.2- and 1.5-fold at 6 and 9 hpi, respectively. At 6, 9 and 12 hpi, the siTroIRF3-treated fish had higher bacterial colony counts in the spleen (15.4-, 3.3- and 4.9-fold). Compared with the control group, the siTroIRF3-treated fish had 9.5- and 2.8-fold higher bacterial loads in the head-kidneys at 6 and 9 hpi, respectively. The PBS-treated group and siTroIRF3-C-treated group (siRNA-C) showed similar bacterial loads, which indicated that the siRNA-C had no effect on the fish’s antibacterial immune responses. Together, TroIRF3 was involved in the antibacterial response against *V. harveyi in vivo*.

### 3.5 Mapping of the NLS domain of IRF3

**Figure 4:**
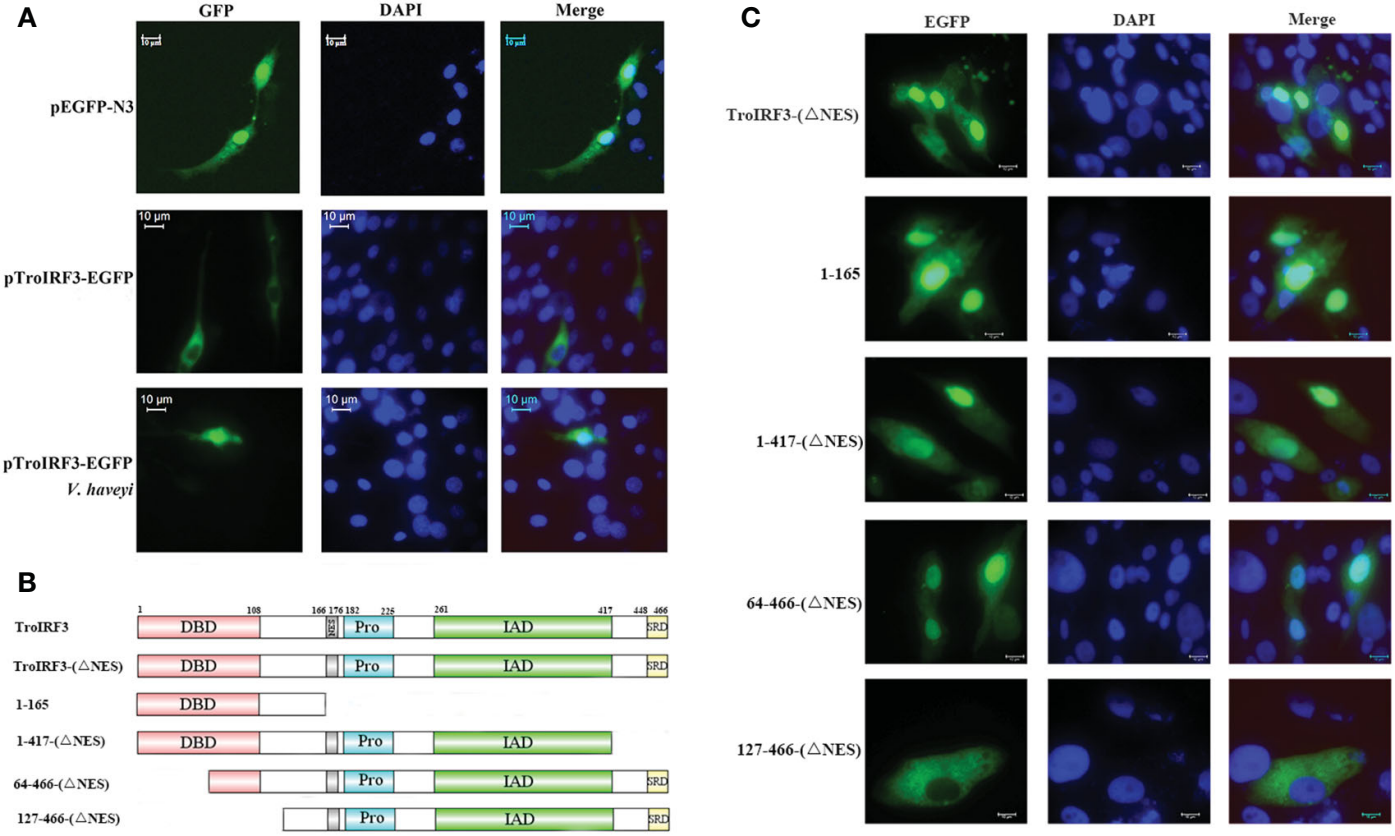
Mapping of the TroIRF3 NLS domain. **(A)** GPS cells were transfected with pEGFP-N3 or pTroIRF3-N3 for 48 h, and the cell transfected with pTroIRF3-N3 were incubated with PBS or *V. haveyi* for six hours, followed by inverted fluorescence microscopy imaging. DAPI was used to stain the nuclei of cells. **(B)** Schematic presentation of TroIRF3-GFP truncation mutants. **(C)** GPS cells were transfected with TroIRF3-GFP truncation mutant plasmids for 48 h before examination by an inverted fluorescence microscope.

The subcellular localisation of TroIRF3 with or without *V. harveyi* stimulation was studied using a fluorescence microscope. EGFP is detected in both cytoplasm and nucleus, TroIRF3 was located exclusively in the cytoplasm in the uninfected cells and translocated to the nucleus after infection with *V. harveyi* (**Figure 4A**). Given the presence of both NLS and NES in IRF3, its predominant cytoplasmic localisation suggested constitutive activities of NES before virus infection, nuclear exports dominated nuclear imports (45). The regulation function of IRF3 was dependent on its nuclear location. To define the TroIRF3’s NLS domain, the NES was inactivated to prevent interference. The protein of TroIRF3 (△NES) was located in the nucleus and cytoplasm of the GPS cells, suggesting that TroIRF3 is capable of entering and staying in the nucleus without NES (**Figure 4C**). To produce different mutants, TroIRF3’s N-terminal and C-terminal regions were truncated (**Figure 4B**). The C-terminal truncations (aa 1–417 and 1–165) and N-terminal truncations (aa 64–466) fused with GFP retained translocation into the nuclei, but the aa 127-466 did not (**Figure 4C**). According to these experiments, the TroIRF3’s NLS domain is located at the N-terminus of aa 64–127.

### 3.6 Mutational analysis of IRF3 NLS

**Figure 5:**
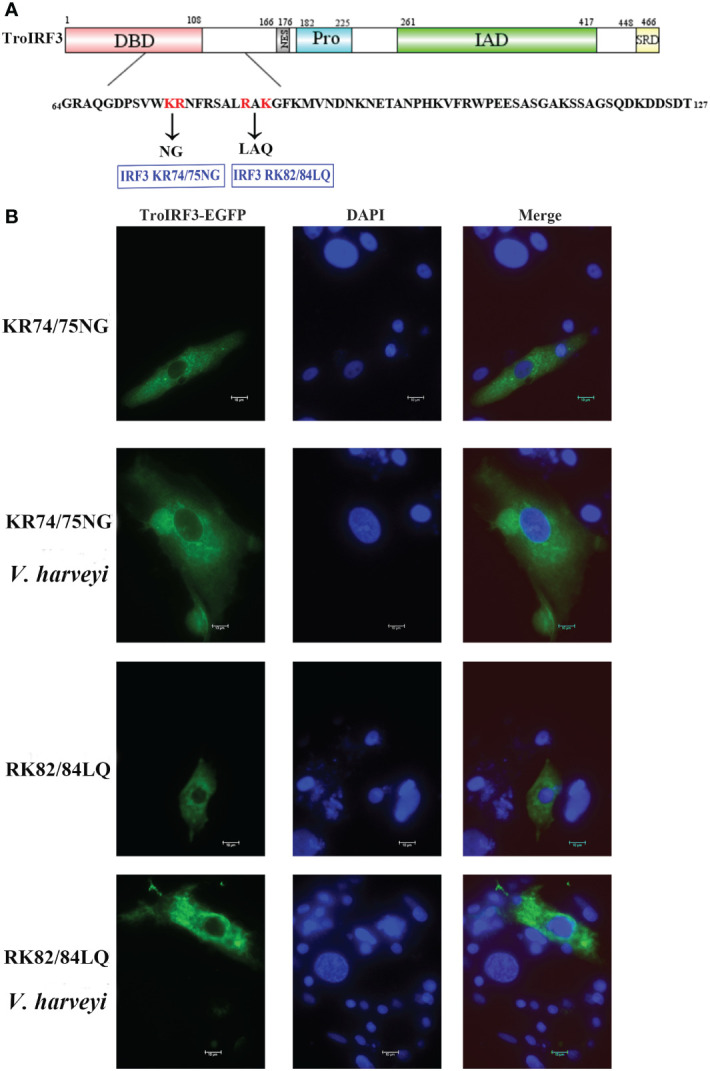
Subcellular distribution of nuclear import of TroIRF3 variants. **(A)** Schematic presentation of different TroIRF3-GFP variants. **(B)** The subcellular distribution of TroIRF3 variants with or without *V. haveyi* infection. GPS cells seeded overnight on a microscope in 24-well plates were transiently transfected with TroIRF3 variants for 48 h. The cells were stimulated by *V. haveyi* for 6 h, followed by inverted fluorescence microscopy imaging. DAPI was used to stain the nuclei of cells.

According to a previous study, mice IRF3 nuclear import requires both basic residue clusters (KR77/78 and RK86/87) (45). The TroIRF3’s NLS domain lies between amino acids 64 and 127. Site-directed mutagenesis was then constructed to determine whether they contribute to the nuclear accumulation of TroIRF3. In TroIRF3 mutants (KR74/75NG and RAK82/84LAQ), basic amino acids (KR74/75 and RK82/84) were replaced with uncharged amino acids (**Figure 5A**). The results of the subcellular localisation assays showed that *V. harveyi* infection caused TroIRF3, but not KR74/75NG and RK82/84LQ, to be localized in the nucleus (**Figures 4A**, **5B**). Therefore, the NLS of TroIRF3 is composed of amino acid residues KR74/75 and RK82/84, which are critical in TroIRF3 nuclear accumulation.

### 3.7 Antibacterial function of TroIRF3 requires NLS

**Figure 6:**
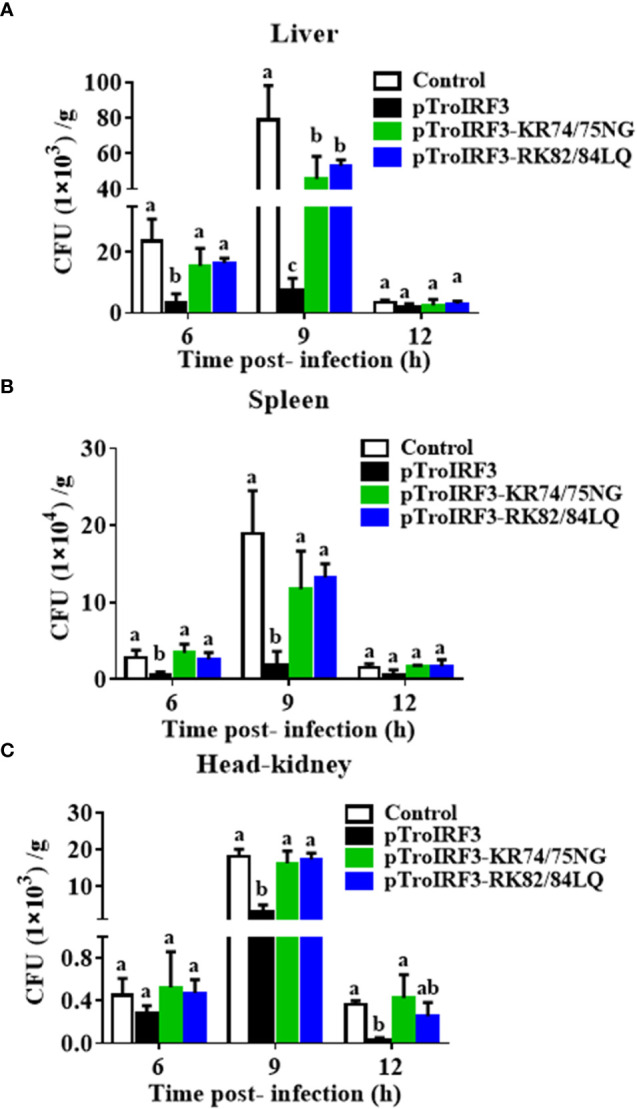
The function of NLS in antibacterial responses regulated by TroIRF3. *T. ovatus* were administered with PBS (control), pTroIRF3, pTroIRF3-KR74/75NG, or pTroIRF3-RK82/84LQ for 5 days, then injected intraperitoneally with *V. harveyi*, and the number of bacteria in the liver **(A)**, spleen **(B)**, and head-kidney **(C)** at different time points were quantified using the plate counting method. Data are expressed as means ± SD (N = 3). N, the number of fish used at each time point per group. Significant differences (*P* < 0.05) are indicated by different letters, and no significant differences are indicated by the same letters.

To determine whether NLS affects TroIRF3-mediated antibacterial defences, the number of *V. harveyi* in the fish treated with pTroIRF3 or the indicated TroIRF3 mutants (pTroIRF3-KR74/75NG and pTroIRF3-RK82/84LQ) was tested. TroIRF3 overexpression significantly suppressed *V. harveyi* replication in the fish, which was in line with our previous observations (**Figure 6**). However, the number of invaded bacteria in pTroIRF3-KR74/75NG- or pTroIRF3-RK82/84LQ-treated fish was enhanced compared with that in the wild-type pTroIRF3-treated fish (**Figure 6**). These results showed that basic amino acids KR74/75 and RK82/84 are required for the efficient antibacterial function of TroIRF3.

### 3.8 TroIRF3-mediated immune gene expression requires an intact NLS

**Figure 7:**
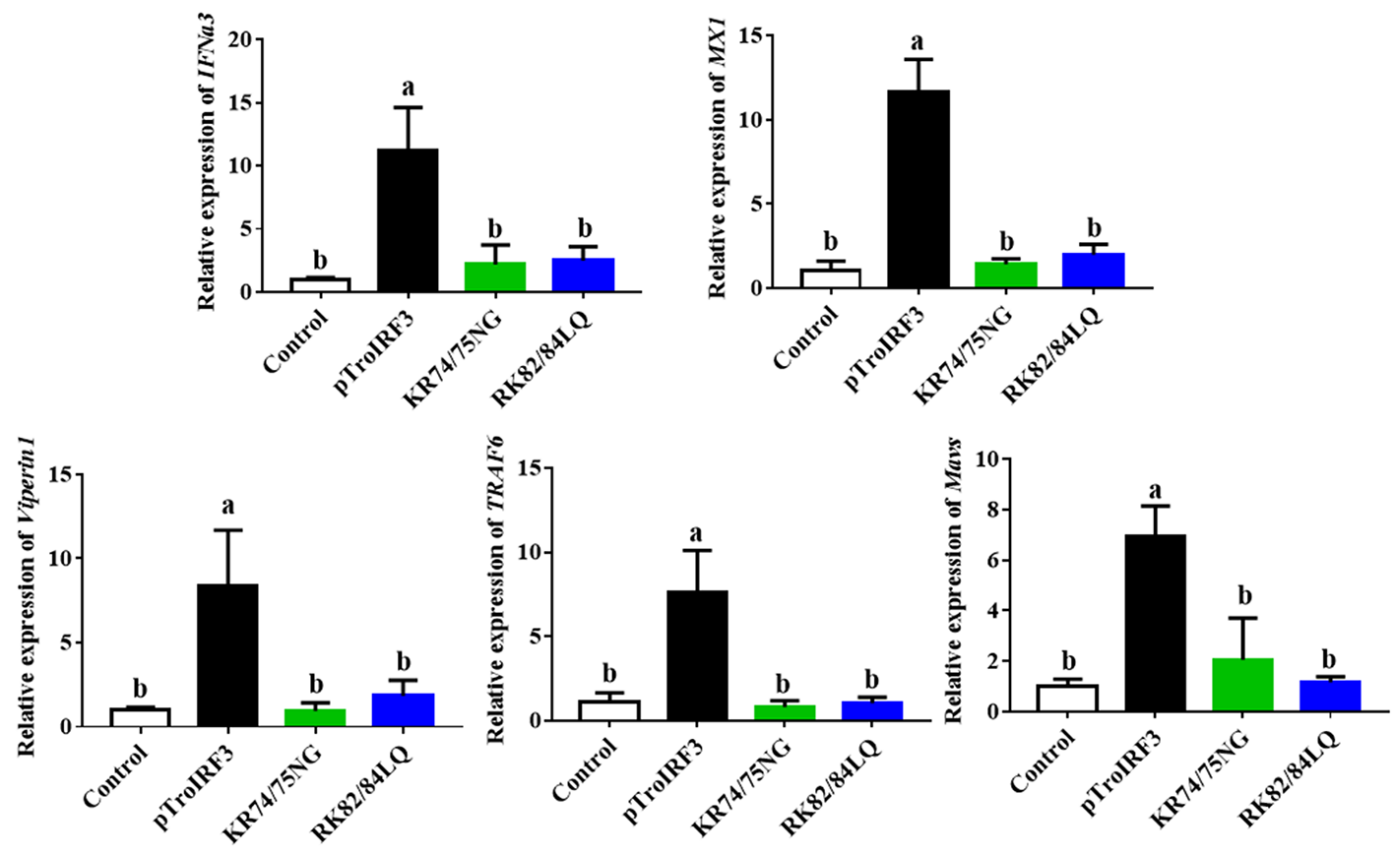
The function of NLS in the expression of interferon signaling molecules induced by TroIRF3. Golden pompano injected with pTroIRF3, pTroIRF3-KR74/75NG, pTroIRF3-RK82/84LQ, or PBS (control). After five days, the expression level of interferon signaling molecules in the spleen was detected. The results represent the mean ± SD (N=3), and N indicates the number of fish used per group. Significant differences (*P* < 0.05) are indicated by different letters, and no significant differences are indicated by the same letters.

To investigate the effects of NLS on the TroIRF3-mediated immune gene expression, the expression levels of immune genes (*IFNa3*, *MXI*, *TRAF6*, *Viperin1* and *MAVS*) in the spleen of the fish treated with pTroIRF3 or the indicated TroIRF3 mutants (pTroIRF3-KR74/75NG and pTroIRF3-RK82/84LQ) were tested. In the pTroIRF3-, pTroIRF3-KR74/75NG- and pTroIRF3-RK82/84LQ-treated fish, the expression level of *TroIRF3* and modified TroIRF3 mutants were significantly upregulated compared with the control fish (**Figure S2**). TroIRF3 remarkably enhanced the production of *IFNa3*, *MXI*, *TRAF6*, *Viperin1* and *MAVS*. However, the expression level of the immune genes (*IFNa3, MXI, TRAF6, Viperin1* and *MAVS*) in the pTroIRF3-KR74/75NG- or pTroIRF3-RK82/84LQ-treated fish was significantly reduced compared with that in the pTroIRF3-treated fish (**Figure 7**). Furthermore, the fish treated with pCN3, pTroIRF3- KR74/75NG or pTroIRF3-RK82/84LQ showed the same expression level of immune genes (**Figure 7**). These results showed that basic amino acids KR74/75 and RK82/84 are required for immune gene induction by TroIRF3.

### 3.9 TroIFNa3 activation mediated by TroIRF3

**Figure 8:**
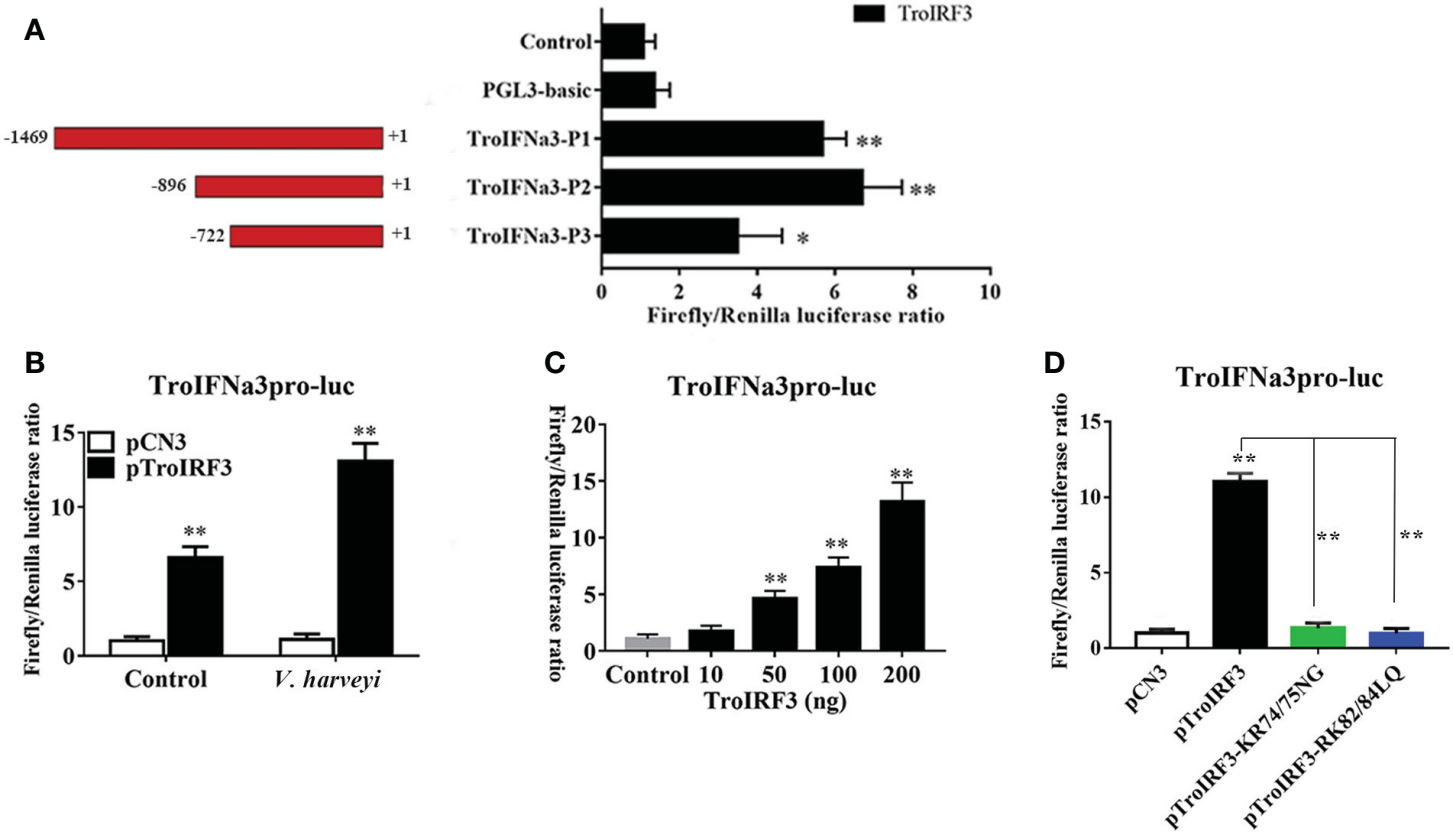
TroIRF3 regulated the activity of the TroIFNa3 promoter. **(A)** Three truncates of the TroIFNa3 promoter were constructed and transfected with pTroIRF3 into HEK 293T cells for 24 h, then harvested for detection of luciferase activity. **(B)** Activation of the TroIFNa3 promoter by TroIRF3. HEK293T cells seeded in 24-well plates were co-transfected with three plasmid sets: pCN3 (0.25 μg), pGL4-TroIFNa3-pro (0.25 μg), and pRL-TK; pTroIRF3 (0.25 μg), pGL4-TroIFNa3-pro (0.25 μg) and pRL-TK (0.025 μg). Cells were stimulated with or without *V. harveyi* after 24 h post-transfection, then harvested for detection of luciferase activity. **(C)** HEK293T cells seeded in 24-well plates were transfected with 0, 10, 50, 100, and 200 ng of pTroIRF3 along with pGL4-TroIFNa3-pro (0.25 μg) and pRL-TK (0.025 μg), then harvested for detection of luciferase activity. **(D)** The effect of NLS on TroIFNa3 promoter activation induced by TroIRF3. HEK293T cells seeded in 24-well plates were co-transfected with TroIFNa3 promoter (0.25 μg), as well as with pCN3, pTroIRF3, pTroIRF3-KR74/75NG, or pTroIRF3-RK82/84LQ (0.25 μg), plus 0.025 mg of pRL-CMV as an internal control. Cells were stimulated with *V. harveyi* for 12 h after 24 h post-transfection, then harvested for detection of luciferase activity. The values are represented as mean ± SD (N = 3). N, parallel experiments. *P < 0.05, **P < 0.01.

Based on the predicted binding sites, consecutive truncated mutants of the TroIFNa3 promoter were constructed to investigate the TroIRF3-binding region in the TroIFNa3 promoter (**Figure 8A**). TroIFNa3-p2 had a higher response to TroIRF3 than the other mutants, suggesting that the region consists of –896 bp to +1 bp from the transcriptional start site, which contains the binding site for IRF3 (**Figure 8A**). The activation of the TroIFNa3 promoter by TroIRF3 was further enhanced by *V. harveyi* stimulation (**Figure 8B**). Furthermore, the activation of TroIFNa3-p2 is dependent on the TroIRF3 concentration (**Figure 8C**).

Subsequently, the potential effects of NLS on TroIRF3-mediated TroIFNa3-p2 activation were investigated using the luciferase reporter assay. TroIRF3 exhibited remarkably enhanced TroIFNa3-p2 activation following *V. harveyi* infection, whereas the other variants (pTroIRF3-KR74/75NG and pTroIRF3-RK82/84LQ) did not exhibit TroIFNa3-p2 activation under the same conditions (**Figure 8D**).

## 4 Discussion

IRF3 activates type I IFN and ISGs, which function as defences against viral and bacterial infections (46). TroIRF3 was identified and characterised in the present study. In addition, the functions of TroIRF3 were investigated, including the intracellular localisation of TroIRF3 and the ability of TroIRF3 to be an activator in antibacterial response and initiator of the transcription of TroIFNa3.

From fish to mammals, the DBD, IAD and SRD domains are conserved in vertebrate IRF3. The DBD domain consists of highly conserved tryptophan residues and forms a helix-turn-helix motif that binds to the ISRE in target promoters (33, 47, 48). After activation, the IRF3 molecules homodimerise through the IAD motif (49). SRD is critical for the phosphorylation IRF3 (12, 14). As with the other IRF3, TroIRF3 has a DBD on the N-terminus and IAD and SRD at the C-terminus. In the phylogenetic analysis, IRF3 from the Osteichthyes species and higher vertebrates cluster into two major groups. TroIRF3 is closely related to *S. lalandi dorsalis* IRF3, which belongs to Perciformes. Given its conserved structure and evolutionary history, TroIRF3 may play a conserved role in the immune response against pathogens.

Studies have found that IRF3 is predominantly expressed in the immune organs of several species, including *Squaliobarbus curriculus* and *E. coioides* (32, 50). In this study, TroIRF3 was most prominent in the gill and liver while relatively low in the spleen and blood. The gill and liver play a role in immunology or act as entry points for pathogens. TroIRF3 showed the highest expression level in gill, which may account for TroIRF3 could be involvement in the early immune response. The spleen and head-kidney from *Odontobutis obscura* also showed relatively low IRF3 expression levels (51). This observation may be attributed to the fact that the mRNA expression check was conducted in healthy fish, where IRF3 was not required for upregulation in the spleen and head-kidney (51). LPS treatment induces IRF3 transcripts in some fish species, such as *L. crocea* and *Ctenopharyngodon idella* (52, 53). *In vivo*, TroIRF3 transcript levels were also significantly upregulated following *V. harveyi* treatment. According to these results, *T. ovatus*’ innate immune response against *V. harveyi* may be mediated by TroIRF3.

In mammals, IRF3 plays a role in antiviral immunity. However, recent studies found that IRF3 also participates in bacterial immunity (54–56). In A549 cells, siRNA inhibiting IRF3 expression resulted in an overall increase in *Legionella* numbers (57). *Chlamydophila pneumoniae* replication was enhanced when IRF3 expression was inhibited by siRNA and attenuated by IFN-β treatment (58). Mice IRF3 deficiency leads to impaired clearance of *P. aeruginosa* from the lung (56). In this study, we showed that the bacterial load reduction in fish tissues was caused by TroIRF3 overexpression. Consistent with these findings, the inhibition of TroIRF3 expression by siTroIRF3 led to an increase in the number of bacteria. In addition, TroIRF3 upregulated key genes, such as IFNa3, MXI, TRAF6, Viperin1 and MAVS, which are involved in IFN/IRF-based signalling. IFN, MXI and Viperin have several functions that participate in the antibacterial and antiviral immune response (59–61). Moreover, TRAF6 and MAVS participate in the activation of IRF3 (62). These findings suggested that the overexpression of TroIRF3 upregulated the expression of IFN and IFN-related genes, leading to an antibacterial state to inhibit *V. harveyi* replication.

Under healthy conditions, mice IRF3 is mostly found in the cytoplasm of cell. After infection with *P. aeruginosa*, it is phosphorylated and transported to the nuclei (56). However, fish IRF3 localization varies. *C. auratus L.* IRF3 was located in the cytoplasm without stimuli but was observed in the nuclei after a poly I: C challenge (33). *Oncorhynchus mykiss* IRF3 is localized in both cytoplasm and nuclei with or without poly I: C and no apparent transfer from the cytoplasm to the nucleus was observed (63). When no bacterial infection is present, TroIRF3 initially resides in the GPS cells’ cytoplasm but undergoes nuclear translocation upon bacterial stimulation. The result suggests that TroIRF3 originally resided in the cytoplasm in an inactive form when no bacterial contamination was present, but when bacteria stimulated the cell, it was activated and translocated into the nucleus. Therefore, TroIRF3 is involved in antibacterial immunity.

IRF proteins contain both NLSs and NESs, so their subcellular distribution can be controlled to manage the IRF function (25). Before bacterial infection, TroIRF3 is predominantly localized in the cytoplasm, suggesting that nuclear export dominates nuclear import. As a result of bacterial infection, TroIRF3 resides mainly in the nucleus, which indicates that nuclear import dominates when TroIRF3 is activated. TroIRF3’s NLS was mapped to aa 64–127, which contains an α-helical pattern. The basic amino acids KR74/75 and RK82/84 are integral to TroIRF3’s nuclear import. KR74/75 and RK82/84 are 90° amino acids apart, which makes a spatial distance of one amino acid between these two clusters that interact with the binding sites in importin-α (**Figure S3**). Similarly, mice IRF3 localizes to the nucleus through a bipartite NLS located at the RK86/87 and KR77/78 sites (45). These results revealed that the amino acids that affected IRF3 nuclear localisation were conserved.

We further analysed the role of NLS in TroIRF3-mediated antibacterial responses and type I IFN expression. Our results showed that KR74/75 and RK82/84 in the NLS of TroIRF3 are involved in antibacterial immunity and immune gene (*IFNa3*, *MXI*, *TRAF6*, *Viperin1* and *MAVS*) upregulation induced by TroIRF3. Similarly, mice IRF3 NLS plays an important role in the IFN response and antiviral immunity mediated by IRF3 (45). Our findings revealed that the NLSs enforced the nuclear localisation of TroIRF3 and were essential to antibacterial immunity in *T. ovatus*.

In DNA or RNA viral infection defence, vertebrate IRF3 is the key transcription factor in the signalling of type I interferon-dependent immune responses (18, 64, 65). Mammalian IRF3 has been shown to bind to the promoters of IFN and ISG and activate their transcription (66). As shown by the luciferase activity, the ectopic expression of TroIRF3 activated the TroIFNa3 promoter. The activation was dependent on the TroIRF3 concentration and further enhanced by *V. harveyi* stimulation. These results may reveal that TroIRF3 can activate TroIFNa3-mediated antibacterial responses. Similar results were also obtained in other species, such as *Miichthys miiuy*, *Lateolabrax japonicus* and *Oreochromis niloticus*. The IRF3 protein of *O. niloticus* enhances the activity of the IFN-β promoter, which plays a role in regulating the IFN response (67). *M. miiuy* IRF3 can activate the IFNα promoter, and the expressions increased with the increase in mmIRF3 quality (68). *L. japonicus* IRF3 overexpression significantly increased the promoter activity of zebrafish IFN1 (69). Furthermore, the amino acid residues KR74/75 and RK82/84 in TroIRF3’s NLS were essential for activating TroIFNa3-p2. These results showed that TroIRF3 acted as a transcription activator in immune responses and that the amino acid residues KR74/75 and RK82/84 are important to the immune regulation of TroIRF3.

In summary, the present study cloned and characterised TroIRF3. *V. harveyi* infection significantly increased TroIRF3 expression and shuttled it from the cytoplasm into the nucleus. TroIRF3 played an important role in the antibacterial immune response. Basic amino acids KR74/75 and RK82/84 were required for the efficient nuclear import of TroIRF3 and played a key role in the antibacterial immune response and upregulation of immune genes induced by TroIRF3. Furthermore, TroIRF3 activated the promoter of TroIFNa3, and the key amino acids KR74/75 and RK82/84 were essential for TroIFNa3-p2 activation induced by TroIRF3.

## Data availability statement

The original contributions presented in the study are included in the article/**Supplementary Material**. Further inquiries can be directed to the corresponding authors.

## Ethics statement

The animal study was reviewed and approved by Animal Care and Use Committee of the Hainan University.

## Author contributions

YS, ZC, YW, and YZ designed the research. PZ, CW, YS, and WY analyzed the data. YW and YZ supervised the study. YS, YW, ZC, and JL performed the research, analyzed the data, and wrote the manuscript with support from all authors. All authors contributed to the article and approved the submitted version.
