# Supplementary material for: IFN regulatory factor 3 of golden pompano and its NLS domain are involved in antibacterial innate immunity and regulate the expression of type I interferon (IFNa3)
Source: Front Immunol. 2023 Feb 2;14:1128196. doi: 10.3389/fimmu.2023.1128196 (PMC9933344; doi:10.3389/fimmu.2023.1128196)
Supplement: Supplementary file 1 [file DataSheet_1.doc]

**Supplement data**

**Table S1**

Primers used in this study.

| Primer name | Sequence(5’-3’) | Amplification target |
| --- | --- | --- |
| TroIRF3-F  TroIRF3-R  TroIRF3-RT-F  TroIRF3-RT-R  siTroIRF3-P1  siTroIRF3-P2  siTroIRF3-P3  siTroIRF3-P4  siTroIRF3-C-P1  siTroIRF3-C-P2  siTroIRF3-C-P3  siTroIRF3-C-P4  B2M-F  B2M-R  IFNa3-RT-F  IFNa3-RT-R  TRAF6-RT-F  TRAF6-RT-R  MXI-RT-F  MXI-RT-R  Viperin1-RT-F  Viperin1-RT-R  MAVS-RT-F  MAVS-RT-R  IFNa3-pF1  IFNa3-pF2  IFNa3-pF3  IFNa3-pR  TroIRF3-F2  TroIRF3-R2  TroIRF3-(NES)-R1  TroIRF3-(NES)-F2  TroIRF3-(NES:1-164)-R1  TroIRF3-(NES:1-443)-R1  TroIRF3-(NES:64-466)-F1  TroIRF3-(NES:127-466)-F1  TroIRF3-KR74/75NG-F1  TroIRF3-KR74/75NG-R1  TroIRF3-RK82/84LQ-F1  TroIRF3-RK82/84LQ-R1 | cccggggccaccATGTCTCATTCCAAACCACTG  cccgggGCACAACTCCATCATCTCTTC  ACAAGAACGAAACCGCTAACCC  TCATCAAAGCACGAGACCACC  GGATCCTAATACGACTCACTATAGCATCA  GGAGCCAAGTCTT  AAAAGACTTGGCTCCTGATGCTATAGTGA  GTCGTATTAGGATCC  GGATCCTAATACGACTCACTATAAAGACTT  GGCTCCTGATGC  AAGCATCAGGAGCCAAGTCTTTATAGTGAG  TCGTATTAGGATCC  GGATCCTAATACGACTCACTATAGCAAGCTG  ACCCTGAAGTT  AAAACTTCAGGGTCAGCTTGCTATAGTGAGT  CGTATTAGGATCC  GGATCCTAATACGACTCACTATAAACTTCAGGGTCAGCTTGC  TATAGTGAGTCGTATTAGGATCCGTGGTGGTGGTGGTGGTG  AAGTCAGTCCACCCAAGGTTCA  GGGATTTCCATTCCGTTCTTCATG  ACACTATGGTCACTACAGCAAC  ACCTCAGTGTTTCGTATGTG  CCCTAAAGCACCCATCGC  AAGGTCACGCAGGAACTCAG  CATACCCTTGGGACCTGA  TGCTTTGGCTTTGTTGAGT  GACCCGTCCAAGTCCATC  CAAAGCCACTGAAGCAAAT  GTTTGGAGGTGCGGATGA  CCTTTTCGGCTTTGCTGTA  CGGGGTACCAAAAGACAACTGATTGTTGA  CGGGGTACCCTGCTACATATAAAAATGT  CGGGGTACCCAATGTGAAGAGGGTTCAG  CCGCTCGAGCATTGACATGATGCCTAACTCT  agatctgccaccATGTCTCATTCCAAACCACTG  gtcgacGCACAACTCCATCATCTCTTC  CTGGTTGATAGTGGATTCTGCT  GCAGAATCCACTATCAACCAGCCTGAATCA  GAGGC  gtcgacCTTTAAATTATCTCCATCGCAG  gtcgacCTGGTTGATAGTGGATTCTGCT  AGATCTGCCACCGGCCGGGCTCAGGGAGAC  AGATCTGCCACCAACCAGCCTGAATCAGA  CCCTCAGTCTGGAACGGGAACTTCCGC  CCCGTTCCAGACTGAGGGGTCTCCCT  GCAGCGCCCTCCTCGCCCAAGGTTTCAAAAT  TTGGGCGAGGAGGGCGCTGCGGAAGTTCC | ORF cloning  qRT-PCR  Synthesizing siTroIRF3  Synthesizing siTroIRF3-C  qRT-PCR  qRT-PCR  qRT-PCR  qRT-PCR  qRT-PCR  qRT-PCR  Cloning IFNa3-pro  Constructing pTroIRF3-N3  Constructing TroIRF3-(NES)  (NES: 1-164)  (NES: 1-443)  (NES:64-466)  (NES:127-466)  Constructing KR74/75NG  Constructing RK82/84LQ |

**Table S2**

Proteins sequences of IRF3 used for phylogenetic analysis.

| Species | GenBank accession number |
| --- | --- |
| *Trachinotus ovatus* | AWY04222.1 |
| *Seriola lalandi dorsalis* | XP_023267113.1 |
| *Siniperca chuatsi* | AVC70699.1 |
| *Lateolabrax japonicus* | AXI69832.1 |
| *Larimichthys crocea* | NP_001290316.1 |
| *Collichthys lucidus* | TKS89890.1 |
| *Miichthys miiuy* | AHB59737.1 |
| *Odontobutis obscura*  *Danio rerio* | QCC62347.1  NP_001137376.1 |
| *Homo sapiens* | XP_024307260.1 |
| *Mus musculus* | NP_058545.1 |

**Fig. S1**


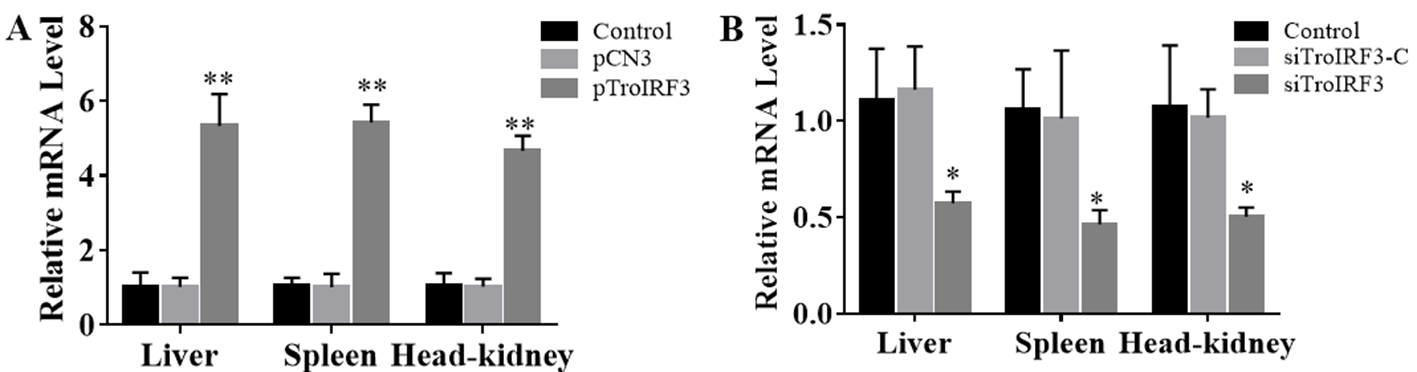


**Fig. S1. Expression of *TroIRF3* in pTroIRF3-treated and siTroIRF3-treated fish.**

*T. ovatus* were injected with PBS, pCN3, and pTroIRF3. At five days post-injection, qRT-PCR was performed to test the expression level of *TroIRF3* in liver, spleen, and head-kidney (A). PBS (Control), siTroIRF3-C, or siTroIRF3 were administered to *T. ovatus*, and qRT-PCR was performed at 12 h post-injection to detect *TroIRF3* expression in liver, spleen, and head-kidney (B). The internal control was *B2M*. At every time point, control fish were set to have an average expression level of 1. Values are shown as means ± SD (N = 3). N, the number of fish used. ***P* < 0.01, **P* < 0.05.

**Fig. S2**

**
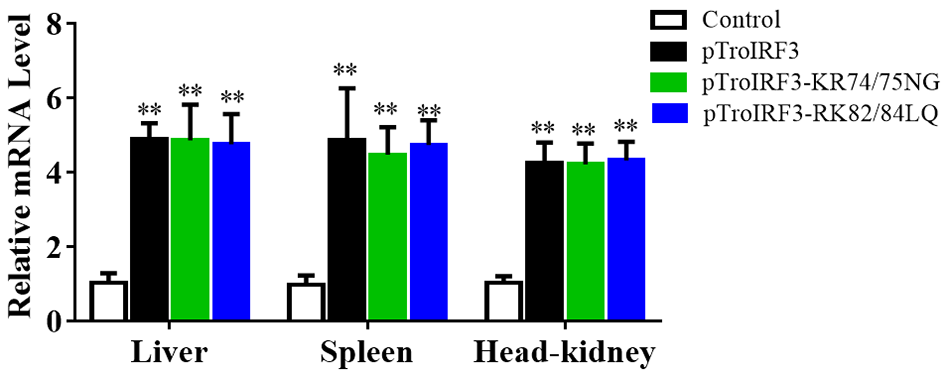
**

**Fig. S2. The expression of *TroIRF3* *and its variants* after plasmids injected in *T. ovatus*.** *T. ovatus* were administered with PBS, pTroIRF3, pTroIRF3-KR74/75NG, or pTroIRF3-RK82/84LQ. The liver, spleen, and head-kidney were collected after five days, RNA was extracted, cDNA was synthesized, and then qRT-PCR was used to detect the expression of *TroIRF3* and TroIRF3 variants. The expression level of the control fish was set to 1. Values are shown as means ± SD (N = 3). N, the number of fish used. **P* < 0.05, ***P* < 0.01.

**Fig. S3**

**
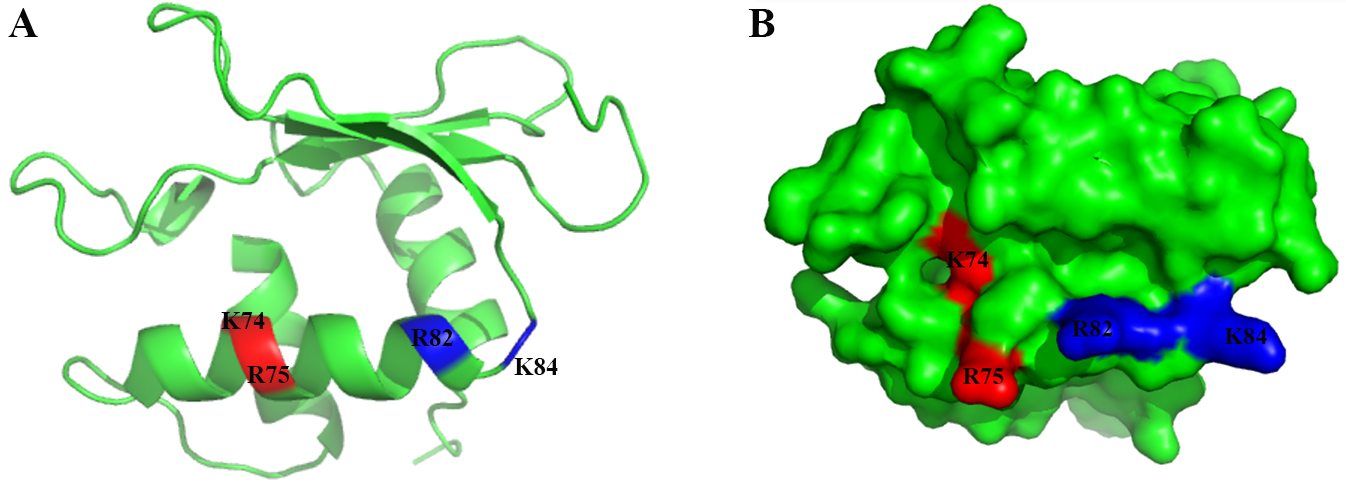
**

**Fig. S3. TroIRF3 NLS domain illustration.** The 3D model of TroIRF3 was built using the structure of IRF3 (SWISS-MODEL Template ID is 2o61.1) as the template.
